# Fracture du col fémoral révélatrice d'un ostéosarcome de la hanche traitée par une prothèse massive

**DOI:** 10.11604/pamj.2016.23.169.8800

**Published:** 2016-04-07

**Authors:** Adil El Alaoui, Fawzi Boutayeb

**Affiliations:** 1 Service de Chirurgie Traumato- Orthopédique A du Centre Hospitalier Universitaire de Fès, Maroc

**Keywords:** Ostéosarcome, hanche, prothèse, Ostéosarcoma, hip, prosthesis

## Image en medicine

Il s'agit d'un patient de 22 ans sans antécédent pathologique notable qui a présenté une semaine avant son hospitalisation des douleurs au niveau de sa hanche droite avec impotence fonctionnelle totale du membre inférieure droit sans notion d'altération d’état général. L'examen clinique fait à l'admission a objectivé une douleur à la palpation et à la mobilisation de la hanche droite. La radiographie standard (A) et le scanner de la hanche (B) ont objectivé une fracture du col fémoral droit sur os pathologique. Une biopsie de la tumeur a été fait revenant en faveur d'un ostéosarcome épiphyso-métaphysaire cervico-céphalique droit. Le bilan d'extension locorégional ne montre pas une autre localisation ou des métastases. Le patient a bénéficié de Sept séances de Chimiothérapie néo adjuvante puis d'une résection carcinologique de la tumeur et une reconstruction par prothèse de hanche massive (C).

**Figure 1:**
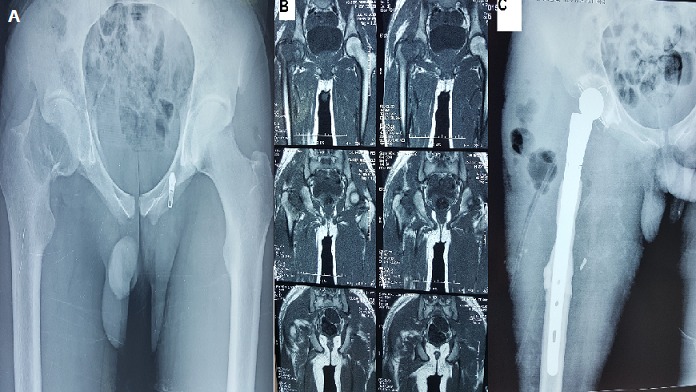
(A) radiographie du bassin objectivant une fracture du col fémoral droit sur os pathologique; (B) scanner du bassin montrant une fracture du col fémoral droit avec une ostéolyse du col et de la tête fémorale; (C) radiographie de contrôle de la hanche droite après la résection tumorale et la reconstruction par une prothèse massive

